# “SO STONED”: Common Sense Approach of the Dizzy Patient

**DOI:** 10.3389/fsurg.2016.00032

**Published:** 2016-06-01

**Authors:** Floris L. Wuyts, Vincent Van Rompaey, Leen K. Maes

**Affiliations:** ^1^Antwerp University Research Centre for Equilibrium and Aerospace (AUREA), University of Antwerp, Antwerp, Belgium; ^2^Department of Biomedical Physics, Faculty of Science, University of Antwerp, Antwerp, Belgium; ^3^Department of Otorhinolaryngology and Head and Neck Surgery, Antwerp University Hospital, Antwerp, Belgium; ^4^Faculty of Medicine and Health Sciences, University of Antwerp, Antwerp, Belgium; ^5^Department of Speech, Language and Hearing Sciences, Faculty of Medicine and Health Sciences, Ghent University, Ghent, Belgium

**Keywords:** SO STONED, history taking, dizzy, vertigo

## Abstract

The history taking of a dizzy patient is of utmost importance in order to differentiate the possible etiologies of vertigo. The key factors that allow a first approximation of diagnosis identification are based on the time profile, symptom profile, and trigger profile of the disease. Here, the proposed mnemonic “SO STONED” comprises eight different dimensions that characterize the vertigo-related complaints of the patient and guide the clinician in his or her decision scheme. All the letters “SO STONED” have a specific meaning: Symptoms, Often (Frequency), Since, Trigger, Otology, Neurology, Evolution, and Duration. Since the most common vestibular diseases have different fingerprints when all dimensions are considered, this tool can facilitate the identification of the appropriate vestibular diagnosis.

## Introduction

Patients with vertigo and dizziness represent a relatively large group in our society with a 7.4% lifetime prevalence (1). Targeted history taking, clinical bedside examinations, functional testing of the vestibular system, and imaging are instrumental for correct diagnosis and therapy management.

For patients presenting with acute vertigo symptoms at the emergency unit, a 2-min, three-item bedside oculomotor examination [head impulse test, nystagmus, skew test (HINTS)] has been proposed by Kattah et al. (2) to differentiate between acute peripheral vestibular lesions or central pathologies, such as cerebellopontine angle infarctions.

Once stroke is excluded, it remains a challenge to identify the etiology of the vertigo. Hereto, clinicians need a quick and comprehensive assessment tool, comprising a structured overview of all the indispensable history information. Based on the literature and our own experience, we made an inventory of how the most common vestibular disorders manifest themselves. This revealed a set of eight independent but closely related dimensions necessary to discriminate between several diagnoses. The acronym “SO STONED” is a useful tool to memorize these eight questions. Recently, Brandt et al. (3) and Roland et al. (4) proposed a similar concept, without however putting it in an “aide memoire” tool, neither covering all our proposed dimensions. The SO STONED tool increases the likelihood of a proper differential diagnosis, which obviously needs to be confirmed by clinical, laboratory, and imaging examinations.

## Proposed Framework

The letters of the acronym “SO STONED” represent: Symptoms, Often (frequency), Since, Trigger, Otology, Neurology, Evolution, and Duration. Table 1 provides an overview of the different questions that should be asked to the dizzy patient. Table 2 represents typical characteristics of different diseases according to the SO STONED history. Classification of the vestibular diseases was based on the ICD-11 beta version of the WHO classification, which divides vertigo in three different categories, namely acute, episodic, and chronic. Since posterior fossa stroke can manifest itself as acute vertigo, stroke was added to the table of diagnoses.

**Table 1:** **Overview of the SO STONED dimensions and their appropriate questions**.

SO STONED			
	Dimensions	Questions	Targeted additional questions
S	SYMPTOMS	What are the symptoms?	Vertigo, dizziness, nausea, postural instability, falls without syncope, falls with syncope, lightheadedness, rotatory or linear sensations, tilt of the vertical, oscillopsia, drunken feeling, lateropulsion, to-and-fro rocking,…
O	OFTEN	How often does “it” happen?	Daily (once or several times a day), weekly, monthly, irregularly, continuously, only once, only during the trigger
S	SINCE	Since when do you suffer from this problem? Both related to time and circumstances	A day, week, month, year, decade ago
			After a viral illness, a head trauma, a medical/surgical intervention, a journey on a boat, train or plane, without any clear cause
T	TRIGGER	What triggers the complaints/symptoms and what makes them worse, i.e., aggravating factors?	General head movements, walking, rolling over in bed, bending over, looking up, being a passenger in a car or plane, walking in supermarket aisles, walking in semi-darkness, coughing, noise, visual stimuli, or … completely spontaneous
O	OTOLOGY	Do you experience any concomitant otological symptoms and when do these occur?	Hearing loss (fluctuating), tinnitus, aural fullness, hyperacusis, autophonia, draining ear, otalgia
			Before, during, or after the attacks, in between, long-lasting, i.e., independent of the symptoms
N	NEUROLOGY	Do you experience any concomitant neurological symptoms?	Headache, migraine (current and in the past), face or limb paresthesia, scotoma, phonophobia, photophobia, numbness, palpitations, hyperventilation, speech problems, diplopia, cervical problems
E	EVOLUTION	What is the evolution of the symptoms?	Persistent, improving, worsening, ups, and downs
D	DURATION	What is the duration of the symptoms?	Seconds, minutes, hours, days, continuously

**Table 2:** **Frequent vestibular disorders characterized by the SO STONED dimensions**.

Category	Pathology	S Symptoms	O Often	S Since	T Trigger	O Otology	N Neurology	E Evolution	D Duration
ACUTE VERTIGO	Vestibular neuritis (VN)/Labyrinthitis (LAB)	Vertigo, nausea, postural instability	Only once	Infection, unknown	Spontaneous, head movement	None, hearing loss (LAB)	None	Recovery over days to weeks	Vertigo + nausea: days
									Postural instability: weeks, months, years, continuously
	Stroke (cerebellar, brainstem)	Vertigo, dizziness, nausea, vomiting, head motion intolerance, postural instability, ataxia	Only once	Sudden onset	Spontaneous	Rare	Cerebellar symptoms (ataxia, hampered diadochokinesis, dysarthria, prominent headache, skew deviation,…)	Recovery	Persistent for days to weeks

EPISODIC VERTIGO	Benign paroxysmal positional vertigo (BPPV)	Vertigo, postural instability	Daily during a certain period	Days, weeks, head trauma, idiopathic, inner ear disease, long term immobility	Head movement, position changes, such as bending over, rolling over in bed,…	None	None	Symptoms persist periods from days to weeks	Seconds
	Ménière’s Disease (MD)	Vertigo, nausea, postural instability	Interval of weeks to months	Months, years, variable duration	Spontaneous, caffeine, stress	Hearing loss, tinnitus, pressure, aural fullness, hyperacusis	None	Fluctuating frequency and intensity	20 min to 24 h
	Vestibular migraine (VM)	Vertigo, dizziness, postural instability	Daily, weekly, monthly, variable	Months, years, history of migraine	Spontaneous, head movement, visual stimuli, sound, fatigue, menstrual cycle, physical exertion	Tinnitus, aural fullness	Headache/migraine, photophobia, phonophobia, scotoma, visual aura	Fluctuating frequency and intensity	Minutes, hours, days (criterion: 5 min to 72 h)
	Vestibular paroxysmia (VP)	Vertigo, dizziness, postural instability	Daily (up to several times)	Variable	Spontaneous, position changes, hyperventilation	With or without: hearing loss, tinnitus, pressure	Paresthesia, headache	Increasing frequency and intensity	Seconds – minutes
	Semicircular canal dehiscence (SCD)/perilymphatic fistula (PF)	Vertigo, oscillopsia, postural instability	Daily, weekly, trigger dependent	Unknown, trauma, congenital, post middle ear surgery, infection (PF)	Pressure, noise, coughing	Hearing loss, tinnitus, pressure, aural fullness, hyperacusis for bodily sounds, autophonia	None	Stable course	Seconds – trigger dependent

CHRONIC VERTIGO	Persistent postural perceptual dizziness (PPPD)	Dizziness, lightheadedness, postural instability	Daily, weekly	Months, preceding vestibular disorder, idiopathic	Spontaneous, sudden movement, visual stimuli, sitting upright	None	Panic attacks	Often progressive	Minutes – hours – days – weeks – continuously
	Bilateral vestibulopathy (BVP)	Dizziness, postural instability oscillopsia, disturbed spatial memory, and navigation	Continuously	Idiopathic, ototoxic medication, infection, bilateral MD, autoimmune disease, DFNA9	Head movement, deprived of visual and/or proprioceptive inputs	None, hearing loss (MD, DFNA9), tinnitus (MD)	None	Slow progression or stable course	Seconds after triggers, continuously

The SO STONED tool proposes a systematic approach to the vertigo patient by using the complementary information provided by the different dimensions. Besides the frequent diagnoses presented in Table 2, other less common diagnoses should be taken into account when appropriate. In the following, we provide the rationale of the different dimensions.

### S: Symptoms

Characterization of the symptoms helps to locate the problem. *Vertigo or spinning*, the illusionary sensation of rotation is primarily referring to semicircular canal-related problems, such as vestibular neuritis (VN), benign paroxysmal positional vertigo (BPPV), superior canal dehiscence, Ménière’s disease (MD), etc. However, vertigo is also a common symptom in stroke (2). *Postural instability* is mainly typical for bilateral vestibulopathy (BVP) or neurologic disorders, such as polyneuropathy or cerebellar ataxia, although it can occur in other pathologies (see Table 2) (5). *Lightheadedness and or dizziness* can be caused by panic attacks, agoraphobia and the formerly called phobic postural vertigo, but recently denominated as “persistent postural perceptual dizziness” (PPPD) proposed by the Barany Society and included by the World Health Organisation (WHO)^1^ in the coming ICD-11 classification. Dizziness is also reported in vestibular migraine (VM), vestibular paroxysmia (VP), BVP, and in stroke (2, 5–7). Some medications can also produce vertigo or lightheadedness. *Linear sensations* or a *tilt of the perceived vertical* are more suggestive for otolith dysfunction (8). Drop attacks or a sensation of being pushed to the ground or in another direction are signs of Tumarkin’s otolithic crisis in MD, but beware of VP, VM or brainstem stroke. *Lateropulsion* as well as *oscillopsia* occur in general when the labyrinth is affected, but may also occur with central lesions, affecting, e.g., brainstem or the vestibulo-cerebellum.

### O: Often

A single attack of acute vertigo is a hallmark of acute peripheral vestibulopathy [VN or labyrinthitis (LAB)], as well as stroke. Attacks that occur almost daily are typical for BPPV, but also for VM, perilymphatic fistulas (PFs), superior canal dehiscence, and VP. When primarily spontaneous attacks occur several times per day, the likelihood for VM decreases compared to VP. MD triggers vertigo attacks more than once, but the symptom-free intervals are rather weeks to months. Chronic vertigo can also be episodic, where the symptoms severity fluctuates in such a way that some complaints are always present as typical for PPPD. Bilateral or inefficiently compensated unilateral vestibulopathy have a more continuous character.

### S: Since

This item focuses on how long the symptoms already exist. Additionally, it aims to identify specific events that occurred in the past and after which all symptoms started. A head trauma occurring some weeks or days before the first symptoms is suggestive for BPPV, but also semicircular canal dehiscence (SCD) or PF can have started with such an event. A viral illness might have preceded VN, which in turn may induce delayed BPPV. Inefficient compensation after an earlier period of vertigo can lead to chronic subjective dizziness, part of PPPD. Ototoxic medication or autoimmune disorders can lead to BVP.

### T: Trigger

This refers to a specific act or situation that provokes or aggravates the symptoms. Bending over, lying down, rolling over in bed, and looking up may all trigger short vertigo attacks, typical for BPPV. It is of great importance to discriminate if the symptoms occur during or just after the lying down or after rising up. Vestibular problems due to BPPV should cause vertigo in particular upon lying down, and often also during the upright movement. If problems only occur during the erecting phase, think of cardiovascular etiologies, such as orthostatic hypotension. Rapid head movements causing instability or dizziness are rather hallmarks for a unilateral or bilateral labyrinth dysfunction, but are also aggravating factors for VM and PPPD. VP is often sensitive to gravity changes, because the irritative blood vessel/vestibulocochlear nerve contact may elicit concomitant vertigo spells lasting several seconds, similar to BPPV. Therefore, when position changes with respect to gravity cause the symptoms, VP should be differentiated from BPPV based on the other dimensions of SO STONED and a clinical investigation of the occurring non-BPPV-like nystagmus.

Besides movement triggers, there are also other types of triggers that can elicit or aggravate vertigo attacks. Valsalva maneuver, coughing, or loud sounds that cause vertigo can be attributed to PFs or a third mobile window lesion, e.g., the superior SCD. Acute periods of stress may trigger MD but also aggravate PPPD. Prolonged sleep disturbances (e.g., stress induced) but also alcohol or exertion can precipitate VM. If visual surroundings cause problems, visual vertigo or VM should be considered. Open squares, shopping malls, and crowded spaces can cause panic attacks that may present with concomitant dizziness, typical for PPPD.

Some vertigo attacks occur completely spontaneously, such as those caused by not only MD, but also VM, VP, and stroke. VN usually starts without a trigger, besides the viral infection, but during the subsequent days each head movement will aggravate the vertigo symptoms. When postural instability is increased in darkness or with unsteady underground, bilateral hypofunction or areflexia could be a differential diagnosis, and in some cases insufficiently compensated unilateral vestibulopathy, particularly when patients are stressed or fatigued.

### O: Otology

Sudden hearing loss accompanied by a single attack of vertigo may suggest labyrinthine deficiency. A combination of tinnitus, hearing loss and pressure in the ears or aural fullness, is suggestive for MD, although other pathologies, such as VM, VP, third mobile window lesions, and also tumors of the internal auditory canal and/or the cerebellopontine angle, such as vestibular schwannomas or meningiomas, may occur with some of the above otological complaints. When autophonia or hyperacusis of bodily sounds (hearing eye ball movements or pulsatile tinnitus) are present, third mobile window lesions should be excluded by means of temporal bone imaging and vestibular evoked myogenic potential measurements.

### N: Neurology

This dimension serves to rule out lesions of the central nervous system, such as posterior fossa ischemia, which could be screened by the HINTS tool (2). Additionally, this dimension aims to identify VM with its typical characteristics, such as headaches, current or past migraine, photophobia, scotoma, phonophobia, paresthesia, diplopia, etc. The absence of specific neurologic symptoms increases the likelihood for peripheral etiologies that present with episodic vertigo attacks, such as MD, BPPV, third mobile window lesions, etc.

### E: Evolution

Vestibular neuritis is typically very disturbing in the early stage with gradual improvement over days, whereas MD is characterized by sudden attacks, with periods of little to no vertigo in between. All other forms of vertigo typically have a stable course of intensity or are characterized by progressive or fluctuating severity of symptoms. One of the rare vestibular disorders that may worsen over time is PPPD.

### D: Duration

The duration dimension has in particular a discriminative power to exclude specific disorders. For instance, MD cannot last seconds to a few minutes, whereas BPPV cannot last hours. Moreover, the dimensions “duration” and “frequency” are closely linked to each other. A single attack, lasting days, can point to LAB or VN, while short but frequent attacks in the order of seconds suggest rather BPPV, VM, VP, PF, or third mobile window lesions. It should be noted that VM can last from seconds (accumulated per day to a total of 5 min) to 72 h (7).

To discriminate between these diagnoses, the combination with the dimension “trigger” and concomitant “neurological” and “otological” signs is crucial. Less frequent attacks that last hours are likely to represent MD or VM. The combination with other dimensions is the key factor using the SO STONED acronym and provides a fingerprint of specific diseases. Clinical examinations, auditory and vestibular tests, and imaging obviously should be done.

## Conclusion

The acronym “SO STONED” is suggested to cover eight different dimensions while taking the systematic history in a vertigo patient. Since the most common vestibular diseases have different fingerprints when all dimensions are considered, this tool can facilitate the identification of the appropriate diagnosis.

## Author Contributions

FW conceptualized and designed the SO STONED tool and drafted the initial manuscript. VR and LM assisted in the interpretation of the SO STONED tool and critically reviewed and revised the manuscript. All authors approved the final manuscript as submitted and are accountable for all aspects of the work.

## Conflict of Interest Statement

The authors declare that the research was conducted in the absence of any commercial, financial, or other relationships that could be construed as a potential conflict of interest.

## Footnotes

^1^World Health Organization, International Classification of Diseases. *ICD-11 Beta Draft*. Available from: http://apps.who.int/classifications/icd11/browse/l-m/en#/http%3a%2f%2fid.who.int%2ficd%2fentity%2f2005792829; https://vestibular.org/pppd#sthash.KWYfrLuT.dpuf
